# Oligochitosan Synthesized by *Cunninghamella elegans*, a Fungus from Caatinga (The Brazilian Savanna) Is a Better Antioxidant than Animal Chitosan

**DOI:** 10.3390/molecules27010171

**Published:** 2021-12-28

**Authors:** Weslley Souza Paiva, Francisco Ernesto de Souza Neto, Moacir Fernandes Queiroz, Lucas Alighieri Neves Costa Batista, Hugo Alexandre Oliveira Rocha, Anabelle Camarotti de Lima Batista

**Affiliations:** 1 Postgraduate Programe in Biotechnology (RENORBIO), Federal University of Rio Grande do Norte (UFRN), Natal 59078-970, Rio Grande do Norte, Brazil; hugo-alexandre@uol.com.br; 2 Laboratório de Biotecnologia de Polímeros Naturais-BIOPOL, Departament of Biochemistry, Federal University of Rio Grande do Norte (UFRN), Natal 59078-970, Rio Grande do Norte, Brazil; moacirfqn@gmail.com (M.F.Q.); lucasalighieri@gmail.com (L.A.N.C.B.); 3 Departament of Medicine, Faculdade Nova Esperança, Mossoró 59628-000, Rio Grande do Norte, Brazil; fernestosn@gmail.com; 4 Biomedicine Departament, Universidade Potiguar, Natal 59056-000, Rio Grande do Norte, Brazil; 5 Department of Agriculture, Federal University of Paraíba (UFPB), Bananeiras 58220-000, Paraiba, Brazil; bellecamarotti@gmail.com

**Keywords:** biomaterials, oxidative stress, antioxidant, calcium oxalate

## Abstract

Animal chitosan (Chit-A) is gaining more acceptance in daily activities. It is used in a range of products from food supplements for weight loss to even raw materials for producing nanoparticles and hydrogel drug carriers; however, it has low antioxidant activity. Fungal oligochitosan (OChit-F) was identified as a potential substitute for Chit-A. *Cunninghamella elegans* is a fungus found in the Brazilian savanna (Caatinga) that produces OligoChit-F, which is a relatively poorly studied compound. In this study, 4 kDa OChit-F with a 76% deacetylation degree was extracted from *C. elegans*. OChit-F showed antioxidant activity similar to that of Chit-A in only one in vitro test (copper chelation) but exhibited higher activity than that of Chit-A in three other tests (reducing power, hydroxyl radical scavenging, and iron chelation). These results indicate that OChit-F is a better antioxidant than Chit-A. In addition, Chit-A significantly increased the formation of calcium oxalate crystals in vitro, particularly those of the monohydrate (COM) type; however, OChit-F had no effect on this process in vitro. In summary, OChit-F had higher antioxidant activity than Chit-A and did not induce the formation of CaOx crystals. Thus, OChit-F can be used as a Chit-A substitute in applications affected by oxidative stress.

## 1. Introduction

Oxidative stress can be divided into three stages: (1) initiation, when the formation of the first reactive species occurs; (2) propagation, when these species, once formed, cause a series of sequential reactions of propagation and production of new reactive species; (3) termination, when the reactive species are converted into stable molecules [1]. Regardless of the stage, humans synthesize or ingest molecules called antioxidants through food that neutralize reactive species and their harmful effects to combat oxidative stress [1].

However, the amounts of these endogenous antioxidants are often insufficient to elicit effective responses against reactive species. In humans, this is compensated by the absorption of exogenous antioxidants by the cells [2]. Therefore, antioxidant molecules are often included in the products of the pharmaceutical, biomedical, and food industries. However, since none of the commercial antioxidants show ideal antioxidant properties, there is a need to find new antioxidants that can adapt to new situations and replace the existing ones [3].

Among all the molecules with potential antioxidant properties, there are polysaccharides [2,3,4]. However, some polysaccharides such as chitosan have low antioxidant activity.

Chitosan is a polysaccharide that is obtained in large quantities annually and is used in the production of various industrial products. This polysaccharide is a linear molecule produced through the chemical deacetylation of the chitin procured from the shell of crustaceans [5] or by the enzymatic deacetylation of the chitin extracted from the cell wall of some fungi [6].

Chitosan originating from the shells of crustaceans, also known as animal chitosan, has applications in several areas, such as environmental [7], pharmaceutical [8], medicine [9], cosmetics [10], and biomaterials [11]. However, the use of animal chitosan has some restrictions, such as the requirement of many chemical compounds for its purification processes, the seasonality in its production, and the allergenicity of the protein residues from the shells of crustaceans [12,13].

Moreover, previous studies have shown that animal chitosan accumulates in the renal tissue [14] and increases the excretion of calcium in urine [15], which is one of the determining factors for the formation of kidney stones. Recent in vitro studies have shown that animal chitosan presence increased the formation of oxalate crystals by approximately 15 times and altered the ratio between CaOx dihydrate (COD) and monohydrate (COM) crystals by 1:4 to 1:2. In addition, the presence of chitosan reduced the size of the COM crystals by approximately 50% and altered the crystal surface charge, making them smaller and less negative and, therefore, more susceptible to adhering to the surface of renal epithelial cells [16,17,18,19,20,21,22,23,24,25,26,27,28].

However, there are no in vivo studies that have evaluated the effect of animal chitosan on the formation of oxalate crystals and, consequently, the formation of kidney stones. These data may further limit the use of animal chitosan.

Fungal chitosan can be used as an alternative to animal chitosan. Chitosan extracted from the cell wall of fungi, particularly those belonging to the class Zygomycetes, do not have these limitations. It is possible to carry out its production on an industrial scale throughout the year in temperature- and pH-controlled environments using inexpensive substrates [17] and smaller amounts of calcium and other ions, thus preventing excess mineral accumulation in humans [18]. In addition, there are no reports on the allergenicity of fungal chitosan due to the absence of allergens such as tropomyosin and arginine kinase in the extraction/production process of this chitosan. These properties make fungal chitosan an alternative to animal chitosan in its many traditional applications [13,19,20].

Companies such as KitoZyme LCC Co. (2020), InvivoGen Co. (2020), and Inbiose NV Co. (2020) produce and manufacture fungal chitosan products for applications in the health sector [5]. Due to characteristics such as low molecular weight, high deacetylation degree, and lower viscosity than crustacean chitosan, fungal chitosan can be an alternative for animal chitosan in healthcare [13,21]. In addition, the search for new fungal species that synthesize chitosan in large quantities and with different characteristics is ongoing.

The Caatinga is an exclusively Brazilian biome located mainly in northeast Brazil. This region has extreme meteorological characteristics such as high solar radiation, high rates of evapotranspiration, average annual high temperatures, low humidity, low rainfall, and low cloudiness, making the biome unique worldwide [22,23].

The fungus *Cunninghamella elegans* is found in the Caatinga, belongs to the order Mucorales, and can be found in the soil, plant-based materials, and other organic substrates [24]. This species can metabolize many compounds through demethylation and oxidation [25]. Moreover, several studies have demonstrated that this species synthesizes chitosan in good quantities, compared with other fungi [26,27,28].

However, there are no data on whether the chitosan from *C. elegans* has the potential to replace animal chitosan in some of its applications. Thus, this study reveals that fungal chitosan has superior antioxidant activity, compared with that of animal chitosan, and does not interfere with the formation of oxalate crystals in vitro tests. These data show that chitosan from *C. elegans* has the potential to be used as an animal chitosan substitute in applications related to oxidative stress.

## 2. Results

### 2.1. Production and Characterization of Fungal Chitosan

From the procedure described in the Methods Section, 20.69 mg of chitosan was obtained from each gram of dry biomass of C. elegans, and its apparent molecular weight was 4.12 kDa. Therefore, this molecule is called oligochitosan. 

**Figure 1:**
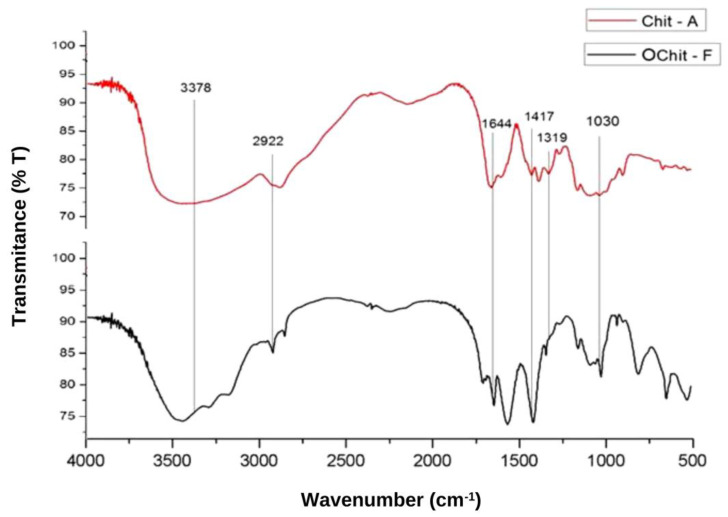
FT-IR spectrum of chitosan from *C. elegans* with the characteristic bands in evidence.

Figure 1 shows the FT-IR spectra of the C. elegans oligochitosan (OChit-F). A large band at approximately 3378 cm^−1^ corresponded to N-H and O-H stretching, and the intramolecular hydrogen bonds. The absorption bands at approximately 2922 and 2877 cm^−^^1^ could be attributed to C-H symmetric and asymmetric stretching, respectively. The presence of residual N-acetyl groups was confirmed by the bands at approximately 1644 cm^−^^1^ (C=O stretching of amide I) and 1319 cm^−^^1^ (C-N stretching of amide III). The small band at 1417 cm^−1^ corresponded to the N-H bending of the primary amine. The absorption band at 1153 cm^−1^ could be attributed to the asymmetric stretching of the C-O-C bridge. The bands at 1066 and 1033 cm^−1^ corresponded to C-O stretching. The same bands were observed in the spectrum of the animal chitosan (Figure 1). This indicates that OChit-F is a chitosan oligosaccharide.

The deacetylation degree (DD%) indicates the number of amine groups in the molecule. The OChit-F DD% was determined using the equation as described in the Methods Section. Therefore, an OChit-F with a 76% deacetylation degree was extracted from the *C. elegans*.

**Figure 2:**
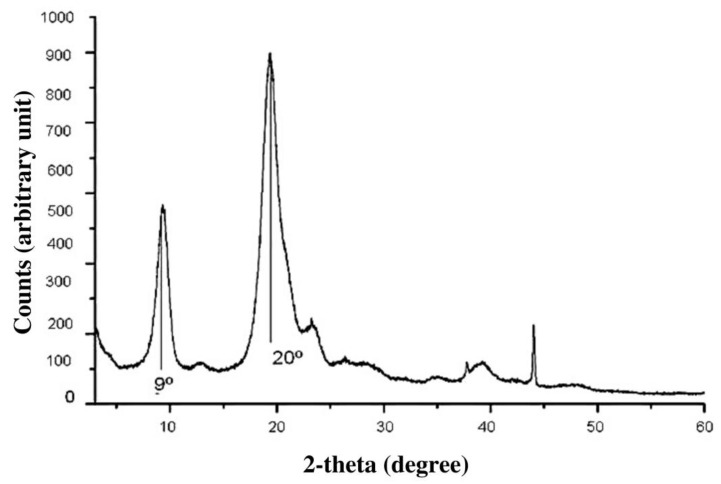
Graph obtained from the structural analysis of the fungal chitosan by X-ray diffraction.

The results of X-ray diffraction of the fungal oligochitosan extracted from *C. elegans* (Figure 2) showed a peak at approximately 20°, corresponding to the crystalline region of the compound, and another at 9°, corresponding to the amorphous chitosan region. 

### 2.2. In Vitro Evaluation of Chitosan Antioxidant Activity

The antioxidant activity of animal chitosan was not superior to that of fungal oligochitosan in any of the tests (Table 1). Fungal oligochitosan showed superior activity in three tests for reducing power, iron chelating activity, and hydroxyl radical scavenging. In addition, the superior activity of the two chitosans determined from the three tests was further verified by the copper chelating test, wherein the two samples showed an activity of approximately 70%. However, there were no significant differences between the two results.

### 2.3. CaOx Formation Assay

#### 2.3.1. Analysis of the CaOx Crystals Profile Formation In Vitro

**Figure 3:**
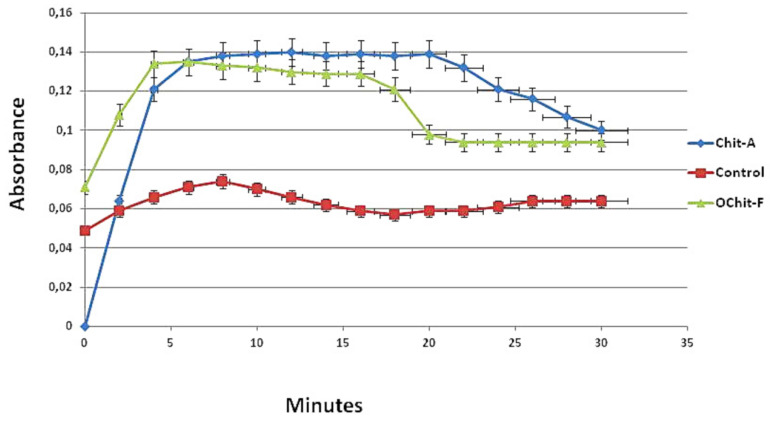
Profile of crystals forming from solutions. Ascending and descending phases of CaOx crystal formation. After 8 min, the formation of the nucleus of the crystals is more intense with Chit-A than that with OChit-F.

The formation profile of calcium oxalate (CaOx) crystals can be divided into two phases: ascending, when there is a constant increase in absorbance due to nucleation and growth of the crystals; descending, when the decrease in absorbance corresponds to the aggregation of the crystals and their subsequent precipitation, which is why the descending phase is known as aggregation. In Figure 3, the light absorption graph of the control group reveals that the nucleation/growth phase reached its peak at approximately 8 min and was subsequently followed by aggregation.

In the presence of animal chitosan, this profile changed. The slope of the curve observed in the nucleation/growth phase was much greater, and the absorbance values were approximately 5 times higher than those observed in the control group. In addition, the period in which the absorbance values remained close to the maximum was much longer (approximately 20 min) than that of the control group (6 min).

The absorption curve obtained in the presence of OChit-F was more similar to that of the control group than the curve obtained in the presence of animal chitosan. The phases of nucleation/growth and aggregation were well defined in the curve prepared with OChit-F. The fall of the curve stabilized in approximately 16 min, showing that the solution balance was established earlier than with Chit-A.

#### 2.3.2. Analysis of Morphology and Quantification of CaOx Crystals by Microscopic Imaging

The following experiments were performed to evaluate the effect of *C. elegans* oligochitosan on the formation of CaOx crystals. 

Initially, the presence of ions in the samples was determined, as the presence of salts could affect the formation of oxalate crystals. Thus, the amounts of Na^+^ and Ca^2+^ were evaluated, in addition to that of K^+^, which can also affect CaOx crystallization.

The results obtained in this analysis showed that the fungal oligochitosan had 0.10 g/kg of Na^+^, 0.0 g/kg of K^+^, and 0.0 g/kg of Ca^2+^. These values revealed that the biopolymer had the minimum amount of Na^+^ and K^+^ or Ca^2+^ residues in its structure. These data confirmed that the changes observed in the CaOx crystallization occurred due to the action of the *C. elegans* oligochitosan and not due to the change in the concentration of these ions in the solutions.

**Figure 4:**
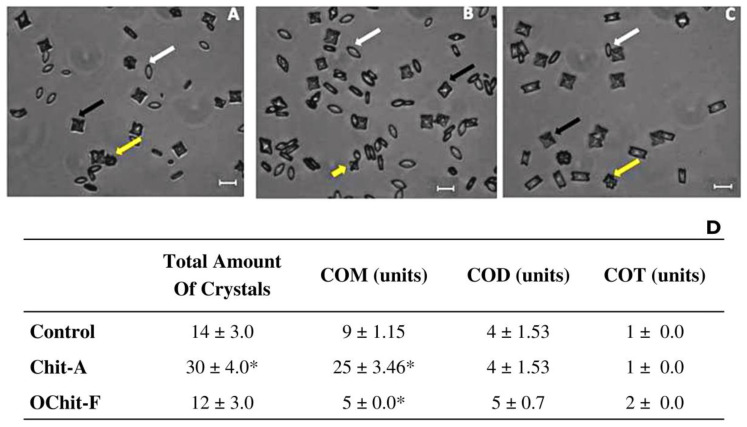
Images of different types of CaOx crystals formed under different conditions. Crystal formation in (**A**) control; (**B**) animal chitosan solution; (**C**) fungal oligochitosan solution. (**D**) Average number of the three types of calcium oxalate crystals, COM, COD, and COT, formed. White arrows (COM crystals), black arrows (COD crystals), and yellow arrows (COT crystals). * Indicates a significant difference (*p* < 0.05) between the crystals formed in the absence (Control CaOx) and presence of samples. The bars correspond to 10 μm. The images were obtained with a brightfield microscope as described in Methods Section.

Calcium oxalate forms three types of crystals in urine: monohydrate (COM), dihydrate (COD), and trihydrate (COT). To the best of our knowledge, no study has evaluated the effect of fungal chitosan or oligochitosan on the formation of CaOx crystals. Therefore, to fill this gap in the related literature, the effect of *C. elegans* fungal oligochitosan on the formation of calcium oxalate crystals in vitro was evaluated. Figure 4A–C depicts the crystals formed under each condition analyzed. In Figure 4D is showed the average number of the three types of calcium oxalate crystals, COM, COD, and COT, formed in the presence of samples, as well as, in control group.

**Figure 5:**
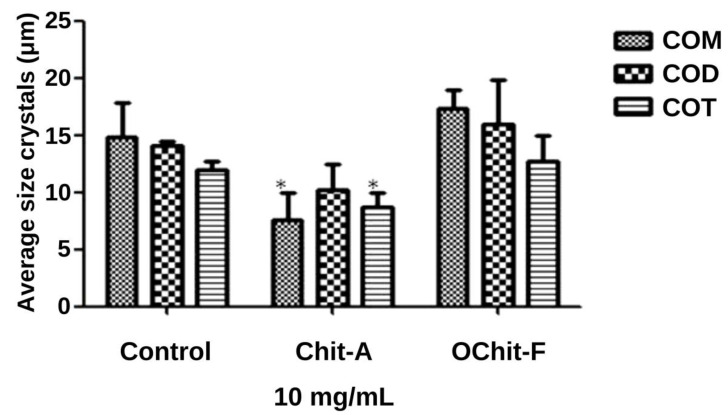
Average size of CaOx crystals formed. * indicate a significant difference (*p* < 0.05) between the crystals formed in the absence (Control CaOx) and presence of samples.

In addition, the size of the crystals was measured as described in the Methods Section, and the data are shown in Figure 5. In control, the sizes of the three types of crystals were COM, 15.0 ± 1.06 µm; COD, 14 ± 1.41 µm; COT, 12.0 ± 0.52 µm. In the presence of animal chitosan, the size of the COT (8.81 ± 0.85 µm) and COM (10.6 ± 0.45 µm) crystals were significantly smaller than those in the control group, whereas the size of the COD crystals did not show any difference, compared with those in the control group. The size of crystals grown in the presence of fungal chitosan was not significantly different from that of those in the control group.

## 3. Discussion

In this study, 20.69 mg of chitosan was obtained from each gram of dry biomass of *C. elegans.* This was lower than the amount obtained with the fungus *Rhizopus arrhizus*, which was 29.30 mg chitosan/g of tissue [29]. However, the amount of chitosan produced by *C. elegans* may differ according to the type of strain used. For example, Berger et al. (2014) obtained 57.82 mg of chitosan per gram of dry mass of *C. elegans* UCP/WFCC 0542 using corn steep liquor as a culture medium [17]. In another study by the same group on a different strain, *C. elegans* UCP 1306, 64.50 mg of chitosan was produced per gram of dry matter. However, in this case, the authors used a substrate different from that used in their first study; they used a medium consisting of cashew juice and whey protein [6].

Although the production of chitosan here was lower than that obtained in previous studies [17,29], further analyses were nevertheless conducted using the obtained chitosan, since the present study aimed to determine the properties of fungal chitosan to enhance its application in the industry. We believe that once this objective is achieved, the next step would be to optimize the large-scale production, extraction, and purification of this chitosan.

According to Wu et al., fungal chitosan with a molecular mass between 1 to 120 kDa is considered to be of a low molecular weight [30]. However, according to reports by Bezrodnykh et al. (2018) [31], chitosan with a molecular mass below 16 kDa is considered to be oligochitosan. Therefore, based on this derivation, our chitosan (4.12 kDa) is referred to as fungal oligochitosan (OChit-F).

Both low-molecular-weight chitosan and oligochitosan are highly valued in healthcare, for example, as a component of carrier molecules to improve the efficiency of controlled drug release [32] and to increase the bactericidal effect of antibiotics against *Helicobacter pylori* [33]. In addition, these chitosans are preferred over chitosan with higher molecular masses because of their greater biocompatibility and biodegradability [31].

According to the European Chitin Society (EUCHIS), a chitin-derivative molecule must have an amine group in more than 60% of its glucosamine residues to be called a chitosan [34]. This concern in determining chitosan DD% is justified because it can modify its applicability. For example, Huang et al. demonstrated that the greater the DD of chitosan is, the greater the absorption of these molecules by fibroblasts is [35]. However, there is a subtle limit, not yet fully understood, that DD can reach, since it has been shown that the closer to 100% the DD of chitosan is, the more difficult it is to degrade and the greater its accumulation in cells is, which, consequently, may trigger toxic effects of chitosan in the tissue [36]. Therefore, some authors recommend that the chitosan DD ideal for medical applications is in the range of 70–85% [36]. Thus, the DD of *C. elegans* oligochitosan was determined using C-N stretching of amide III and N-H bending of the primary amine, which was conducted to identify deacetylation groups in chitosan polysaccharide [29,30,31,32,33,34,35,36,37,38].

In the present study, the DD of *C. elegans* oligochitosan was within the range recommended by Freier et al. [36], and the material extracted from *C. elegans* was confirmed to be an oligochitosan, and not chitin [36]. The DD of the oligochitosan in the present study is superior to that found by Wu et al. [38], who extracted chitosan from the fungus *Agaricus bisporus* of DD 66% and similar to that found by Namboodiri and Pakshirajan [39], who extracted chitosan of DD 77%. Studies with other fungi of the same class as *C. elegans* reported chitosan with different DD, for example, *Cunninghamella elegans* SIS 41, DD 86% [20], and *Rhizopus stolonifer*, DD 84% [18]. Therefore, there is consensus on the fact that the DD of chitosan varies according to the carbon/nitrogen source in which the fungus was grown, which justifies the results obtained for the DD of the chitosan extracted from a *C. elegans* found in [20], which is different from the one described in the present study.

The bands in the FT-IR spectrum of *C. elegans* oligochitosan represent typical polysaccharide characteristics and are found in other polysaccharide spectra [40,41,42], including chitosan spectra [6,29,32,34,43,44,45]. In addition, the peaks shown in Figure 2 (X-ray diffraction) were also found in graphs of other similar analyses of chitosan from both animal [46] and fungal [5] sources. These data provide further evidence that the material obtained from *C. elegans* is essentially chitosan.

Cellular metabolism and stressors can induce the formation of reactive species. These species are chemically unstable and interact with surrounding molecules, inducing the chain formation of new reactive species. When uncontrolled, this process is called oxidative stress and causes damage to cells and tissues. Therefore, it is associated with the manifestation of several conditions such as premature aging [47], rheumatoid arthritis [48], neurodegenerative diseases [49], disorders of anxiety and depression [50], and urolithiasis [51].

The reducing power test evaluates the ability of a molecule to donate electrons. Our data showed that animal chitosan exhibited no activity in all three tests, whereas fungal oligochitosan showed subtle activity only in the reducing power test. The low or no ability to donate electrons of chitosan has already been demonstrated in the literature [52,53], which strengthens the possibility that the ability to donate electrons is not the main mechanism behind the antioxidant action of chitosan. It has been reported that the association between betulinic acid and fungal chitosans in liposomes produces a high antioxidant effect; however, the same association with animal chitosan results in much lower antioxidant activity [30]. Chelating agents are used as nutritional supplements, additives for cleaning chemicals, cosmetics, plastics, fertilizers, toxic metal removers from the soil and body, and antioxidant agents [33]. The capacity of these chelating agents to bind to metals, mainly iron and copper, gives them a good antioxidant capacity, which prevents these metals from oxidizing the cells; hence, it is important to test their metal chelation capacity.

For the iron chelation test conducted in the present study, the samples showed low activity (fungal oligochitosan) or were undetectable (animal). This result differs from that of other studies [53,54,55], which reported good iron chelating capacity for their chitosan (animal or fungal). The difference in results could be due to the different molecular mass and DD, as these factors interfere closely with chitosan activity. The oligochitosan of *C. elegans* has a low molecular weight and has a DD of approximately 76%. According to some authors, to obtain a good chelating capacity, it is important that the chitosan molecule has a low apparent molecular weight but a DD higher than 90% [53,54,55,56].

Copper is also a metal of great importance in oxidative processes. This metal is a fundamental trace element for a series of biological processes, including mitochondrial respiration, antioxidant defense, and neurotransmitter biosynthesis [57]. When there is an imbalance in the amount of free copper in the human body, the emergence of a series of diseases can be triggered, including Alzheimer’s disease, Parkinson’s disease [57], diabetes [58], idiopathic pulmonary fibrosis [59], and cancer [60]. Therefore, it is necessary to study new molecules that may balance the amount of unbound copper in the body, preventing its free ions from triggering cell oxidation and, consequently, leading to the emergence of the diseases mentioned above [61].

The results of copper chelation show that fungal oligochitosan and animal chitosan have good abilities to chelate copper (approximately 70%). Chitosan can interact with Cu^2+^ because of its amine [62] and primary hydroxyl groups [63]. From this information, it can be inferred that the greater the DD and molecular weight of chitosan, the more amine and hydroxyl molecules produced by chitosan, and the greater its ability to chelate copper. A study by Queiroz et al. [16] confirmed this inference by their report on chitosan with a DD of approximately 76%, similar to that found in *C. elegans* oligochitosan, but with a molecular weight about 10 times greater, which showed more than 80% copper chelation.

Vino et al. used sulfated animal chitosan with a DD lower than 60% and failed to achieve copper chelation activity greater than 65%. One explanation for this result is that during the sulfation process, the sulfate binds to the free amine and, consequently, decreases the amount of free amine that can chelate copper [52].

In the hydroxyl radical scavenging test conducted in the present study, fungal oligochitosan showed 40% scavenging activity, which is considered important because the hydroxyl radical (HO) is the most harmful to an organism and has a short half-life, which makes it difficult to scavenge in vivo. This result is in contrast with that reported by Yen et al. [64], which indicated that chitosan from the fungus *Lentinula edodes* showed 77% hydroxyl radical sequestration. In contrast, animal chitosan showed no activity. These results were in contrast with those found by Jing et al. [65], wherein chitosan conjugated with tannic acid had 90% DD and showed 90% hydroxyl radical sequestration. Yang et al. [66], on the other hand, used animal chitosan with 97% DD and achieved 60% hydroxyl radical sequestration. This difference can be explained by the difference in DD between these chitosans, i.e., chitosan more deacetylated has more hydroxyl groups than the chitosan with low DD. Additionally, according to Li et al. [67], the ability to sequester the hydroxyl radical of polysaccharides is associated with free alcohol or hydroxyl groups in its structure. These results corroborate the data from the study by Yen et al. [64], who used fungal chitosan with different degrees of deacetylation (78%, 85%, 90%) that exhibited different hydroxyl radical scavenging (62%, 68%, and 77%), demonstrating a directly proportional relationship between the DD and the capacity to sequester the hydroxyl radical, i.e., the greater the DD, the greater the capacity to sequester hydroxyl radicals.

Figure 3 shows that the presence of chitosan altered the balance between the salts and crystals to the detriment of the latter, which led to an increase in the number of crystals formed. Similar results were previously reported by Queiroz et al. [16]. 

Grohe et al. [68] showed that charged macromolecules, can both positively and negatively regulate the morphology and size of the crystals. However, it is suggested that larger molecules have a higher capacity to interfere with these crystal parameters because when interacting with regions of the crystals, these larger molecules would hinder the continued process of crystal growth due to their size, inducing the formation of smaller crystals than those observed in the control groups [16]. Therefore, it is believed that fungal oligochitosan did not induce the formation of smaller crystals due to its small size. This is important because smaller crystals can be more toxic [69,70].

Some studies have shown that small-sized COM crystals are more toxic because they are more easily phagocytosed, and upon entering the cell, they cause an increase in oxidative stress leading to cell damage and death [69,70]. Thus, the results of this study show that fungal chitosan, unlike animal chitosan, does not favor the increase in the formation of CaOx crystals (mainly COM) and does not reduce the size of the crystals. 

## 4. Materials and Methods

### 4.1. Animal Chitosan

Animal chitosan used in this work was from Polymar ^®^ (Fortaleza, Ceará, Brazil), with a degree of deacetylation = 88% and molecular weight = 50 kDa.

### 4.2. Obtaining the Fungal Strain and Extraction of Chitosan

Soil samples were collected at 10 different points in the ESEC Seridó reserve, located in the southwest region of the state of Rio Grande do Norte, Brazil, between the geographic coordinates 06° 35′ and 06° 40′ south, and 37° 20′ and 37° 39′ west. This conservation unit is in the caatinga biome, a region with high temperatures and low rainfall, with a total extension of 1123.59 hectares and a vast amount of environmental diversity. The sampling areas were randomly selected and marked by GPS. The fungus isolation was performed as described earlier [71]. The identified fungus is deposited cataloged in the Culture Bank of the Catholic University of Pernambuco, Recife, Pernambuco, Brazil.

After the strains had been isolated and identified, the spores were collected and stored in 15 mL of sterile distilled water (standard solution). About 10^5^ spores/mL of the standard solution were added in 400 mL of YPD medium (Yeast Extract 10 g; Peptone 20 g; Dextrose 20 g per liter) and incubated at 28 °C/96 h in static mode. Biomass was filtered, lyophilized, and chitosan was extracted according to [72], with some modifications as described [73,74]. 

### 4.3. Physical–Chemical Characterization of the Chitosan Molecule

#### 4.3.1. Molecular Weight Determination

The molecular weight of fungal chitosan was determined by size-exclusion chromatography. Ultrahydrogel columns 500 and 250 with 7.8 × 300 mm (Waters Corp., Milford, MA, USA) were connected in series to an Accela^®^ HPLC (Thermo Scientific, Waltham, MA, USA). The eluent was filtered (0.22 m membrane) pure water with 0.1 M sodium nitrite (NaNO2) at a flow rate of 0.6 mL/min at 30 °C. A set of dextran standards (6, 10, 40, 71, and 147 kDa) was purchased from Sigma-Aldrich (St. Louis, MO, USA), used to construct the standard curve for the determination of the molecular weight.

#### 4.3.2. Fourier Transform Infrared Spectra (FT-IR)

The spectra were obtained over a frequency range of 500 and 4000 cm^−^^1^ using a Nexus 470 ESP FT-IR spectrometer (Thermo Nicolet, Madison, WI, USA), of a tablet containing mixed KBr and different samples (5 mg). In total, 32 scans at a resolution of 4 cm^−^^1^ were evaluated and referenced against air according to [37]. The deacetylation degree (DD%) of fungal chitosan was determined by FT-IR. To determine DD%, the following equation was used: A1320/A1420 = 0.3822 + 0.03133 [37]. 

#### 4.3.3. X-ray Diffraction 

X-ray diffraction measurements were performed using a diffractometer Shimadzu XRC6000, Kyoto, Japan, with copper tube (l = 1.54 Å); the voltage and current used were 40 kV and 30 mA, respectively. These measurements were performed in the range of 3–50 °C with a scanning rate of 1°/minute in steps of 0.02°.

#### 4.3.4. Ions Determination 

The determination of Na^+^, K^+^, Ca^2+^ metal ions was carried out at the Soil Analysis Laboratory, Universidade Federal Rural do Semi-árido (UFERSA), Mossoró, RN, Brazil. Na^+^ and K^+^ were measured in a flame photometer Digimed DM-62, São Paulo, Brazil, and Ca^2+^ by atomic absorption spectroscopy, Agilent AA240FS, Santa Clara, CA, USA.

### 4.4. Antioxidant Activity In Vitro

#### 4.4.1. Iron Chelating Activity

The method used ferrozine and the FeCl_2_ complex to determine the antioxidant capacity, as described in [75]. The samples (initially 10 mg/mL) were added in different concentrations (0.1 to 2.0 mg/mL), along with FeCl_2_ (2 mM) and ferrozine (5 mM). Then, the mixture was homogenized. After 10 min of incubation at 37 °C, the absorbance was determined at 562 nm on a microplate reader.

#### 4.4.2. Copper Chelating Activity

The copper chelating ability from the samples was determined according to the method described in [76]. The test was performed on 96-well microplates using a reaction mixture containing different sample concentrations (0.1–2 mg/mL), pyrocatechol violet (4 mM), and copper (II) sulfate pentahydrate (50 mg/mL). All wells were homogenized with the aid of a micropipette, and the absorbance of the solution was measured at 632 nm.

#### 4.4.3. Reducing Power Test

The reducing power of the samples was examined according to Melo-Silveira et al. [75]. Briefly, different polysaccharide concentrations (0.05–1.0 mg/mL) were added to a solution of 200 mM sodium phosphate buffer (pH 6.6) and potassium ferricyanide (10 mg/mL). After incubation at 50 °C for 20 min, trichloroacetic acid (10% *w*/*v*) and ironIII chloride (0.1% *w*/*v*) were added. The mixture was stirred, and the absorbance (700 nm) was measured using a microplate reader. Results were expressed as the percentage of activity observed for 0.1 mg/mL (highest activity) of ascorbic acid.

#### 4.4.4. Hydroxyl Radical Scavenging Assay

Hydroxyl radical scavenging assay was determined according to the method described earlier [75]. To perform this test, the hydroxyl radical was generated using 3 mL of sodium phosphate buffer (150 mM, pH 7.4), containing 10 mM FeSO_4_·7H_2_O, 10 mM EDTA, 2 mM sodium salicylate, 30% H_2_O_2_, and different concentrations of the polysaccharides. The radical was generated via Fenton reaction (Fe^2+^ + H_2_O_2_ → Fe^3+^ + OH^−^ + OH). For the control, phosphate buffer was used instead of hydrogen peroxide. After incubation at 37 °C for 1 h, the absorbance of the presence of hydroxyl radical was measured at 510 nm. Gallic acid was used as standard.

#### 4.4.5. Total Antioxidant Capacity (TAC)

Total Antioxidant Capacity (TAC) was carried out according to Melo-Silveira et al. [75]. It is based on the reduction of Mo (VI) to Mo (V) by the sample, followed by the formation of a green phosphate/Mo (V) complex at acidic pH. Tubes containing chitosan and reagent solution (0.6 M sulfuric acid, 28 mM sodium phosphate, and 4 mM ammonium molybdate) were incubated at 95 °C for 90 min. After incubation and the mixture had cooled to room temperature, the absorbance of each solution was measured at 695 nm against blank. The total antioxidant capacity was expressed using ascorbic acid as a standard comparison.

### 4.5. CaOx Formation Assay

#### 4.5.1. Crystallization of Calcium Oxalate (CaOx)

This test was carried out as described earlier [42]. In this test, a mixture of calcium chloride (8 mmol/L), sodium oxalate (1 mmol/L), sodium chloride (200 mmol/L), and sodium acetate (10 mmol/L) (control solution) was prepared. Thus, crystallization was induced by the addition of Na_2_(CO_2_)_2_. The concentrations of this mixture are close to the physiological concentrations of urine. The formation of the crystals was evaluated in the presence and absence of animal chitosan and fungal chitosan. From the absorbance values obtained, it was possible to create a graph that represents the profile of CaOx crystal formation in the presence of the chitosan sample [42].

#### 4.5.2. Analysis of the Morphology of CaOx Crystals by Microscopic Image

Crystals were formed in the presence and absence of the polysaccharide. After formation, the solutions with crystals were centrifuged at 5000× *g* and the supernatant was discarded. The precipitate is composed mainly of CaOx crystals and was resuspended in 0.5 mL of water. A 0.1 mL aliquot was placed on a histological slide and observed under an optical microscope (NIKON ECLIPSE Si, Melville, NY, USA) (400×) immediately after resuspension. Images were obtained from 10 different fields, randomly selected, from each slide, and were analyzed using the NIS Elements AR 4.00.03 64-bit software, Nikon (2011), Melville, NY, USA. Consequently, the number and size of CaOx were determined. Three independent experiments were performed, each with three replicates.

### 4.6. Statistical Analysis

The PAST program, Hammer version 2.17c (2013), Oslo, Norway, was used. An ANOVA test was performed, which measures the variability between the values. Subsequently, the Tukey test was applied to assess whether there was a significant difference between the means generated with the experiments.

## 5. Conclusions

Structural analyses by FT-IR and DRX indicated that the biomaterial extracted from *C. elegans*, isolated from the soil of the Caatinga biome, is chitosan with a 76% deacetylation degree. This material has a low molecular weight and is known as fungal oligochitosan-OChit-F. Chit-A promoted an increase in the number of crystals, mainly in the form of COM, and decreased their size. On the other hand, OChit-F showed no increase in the formation of crystals or altered their sizes. In addition, Ochit-F showed hydroxyl radical scavenging activity superior to that of Chit-A and exhibited iron-chelating and reducing-power activities, which were absent in Chit-A.

In summary, the results show that OChit-F is a promising antioxidant agent that does not affect CaOx crystal formation. Therefore, OChit-F has the potential to be used as a Chit-A substitute in several applications. 

## Figures and Tables

**Table 1:** Antioxidant activities of fungal oligochitosan (OChit-F) and animal chitosan (Chit-A).

	OChit-F	Chit-A
Total antioxidant capacity (TAC) *	ND	0.33 ^a^
Iron chelation	13% ± 1.0 ^a^	ND
Copper chelation	70.3% ± 1.2	70.7 ± 3.2
Reducing power	17% ± 1.0 ^a^	ND
Hydroxyl radical scavenging	40% ± 1.0 ^a^	ND

ND—Not detectable until 2.0 mg of sample was tested. * Each gram of the sample had an activity in the TAC test similar to that of 0.33 mg of ascorbic acid. ^a^ Significant difference was indicated by *p* < 0.001.

## Sample Availability

Samples of the compounds are available from the authors.
